# Donnan Membrane Process for the Selective Recovery and Removal of Target Metal Ions—A Mini Review

**DOI:** 10.3390/membranes11050358

**Published:** 2021-05-14

**Authors:** Dennis Asante-Sackey, Sudesh Rathilal, Emmanuel Kweinor Tetteh, Elorm Obotey Ezugbe, Lingham V. Pillay

**Affiliations:** 1 Department of Chemical Engineering, Durban University of Technology, Durban 4001, South Africa; ingsackey@gmail.com (D.A.-S.); rathilals@dut.ac.za (S.R.); elormezugbe.ee6@gmail.com (E.O.E.); 2 Department of Process Engineering, Stellenbosch University, Matieland 7600, South Africa; pillayvl@sun.ac.za

**Keywords:** Donnan membrane process, ion exchange membranes, metal recovery, Donnan Dialysis

## Abstract

Membrane-based water purification technologies contribute significantly to water settings, where it is imperative to use low-cost energy sources to make the process economically and technically competitive for large-scale applications. Donnan membrane processes (DMPs) are driven by a potential gradient across an ion exchange membrane and have an advantage over fouling in conventional pressure driven membrane technologies, which are gaining attention. DMP is a removal, recovery and recycling technology that is commonly used for separation, purification and the concentrating of metals in different water and waste streams. In this study, the principle and application of DMP for sustainable wastewater treatment and prospects of chemical remediation are reviewed and discussed. In addition, the separation of dissolved metal ions in wastewater settings without the use of pressure driven gradients or external energy supply membrane technologies is highlighted. Furthermore, DMP distinctive configurations and operational factors are explored and the prospects of integrating them into the wastewater treatment plants are recommended.

## 1. Introduction

Metals, specifically heavy metals in effluent and sludge discharges from anthropogenic sources such as households, agriculture, manufacturing and process industries, are of major concern to environmental regulators [1,2,3]. Notable amongst the metals and those that are classified as the most hazardous metal species are As, Cr, Ni, Cd, Pb, Co, Zn and Cu. Although the concentration of these metals very depending on the source, they are toxic and non-biodegradable, even at very low concentrations. Due to the high solubility of these metals, they are readily passed-on, absorbed and accumulated into the human body through the food chain, thereby causing cancers, neurological disorders, skin diseases, respiratory problems, congenital disorders, fertility decreases and chronic kidney damage [4,5,6].

Knowing the aforementioned impact on water, soil and air, public concerns have increased over the years resulting in stricter legislations, most especially in more developed countries [7]. However, various management and control schemes to address the adverse effects at their point sources and non-point sources have not achieved the extent of impact. While the presence of the metals in the discharges have been viewed as toxic and require complete removal, new age engineering considers them as a representation of a significant loss in raw materials. Sustainable treatment options in addressing the latter view, therefore, look at removal, recovery and reuse technologies (3Rs-Tech).

Ion exchange for the removal, recovery and reuse of metals is a widely known and effective treatment process. It is a selective, reversible and stoichiometric method that involves the displacement of ionic species by another ionic species in the exchanger [8]. The exchangers serve as sorbents and are either resins or membranes. Although the mention of ion exchange usually refers to resins, ion exchange membranes (IEMs) have gained prominence due to their dimensional stability over resins [9]. Wide spread use of IEMs include sea water desalination, water softening and purification, the chlor-alkali process, energy production and energy storage [10,11,12].

The Donnan membrane process (DMP), commonly referred to as Donnan Dialysis, is an emerging green treatment process that integrates IEMs. The first usage of DMP is attributed to Prakash and SenGupta [13]. The DMP involves the stoichiometric counter transport of ions across an IEM. As a concentration gradient driven process, DMP can be classified as a 3R-tech used in the recovery, separation and concentration of ions of interest from diluted solutions.

The DMP has often been interchanged with Diffusion Dialysis (DD) due to their indistinguishable principles of operation and application advantages. Whilst DD is utilized in the recovery of mineral acids or alkalis from waste acid and alkaline solutions, DMP is applied in the recovery of toxic or valuable heavy metal ions [14,15,16]. The simple and easy to operate DMP system exhibits functional advantages over the conventional ion exchange process, electrodialysis (ED), chemical precipitation and pressure driven membrane processes. The DMP is an energy efficient, low installation and operational cost, non-risen regeneration and a non-fouling process that possess rural application benefits [17,18,19,20,21]. Ion transport in a DMP occurs as long as the donor phase volume is greater than the receiver phase. Table 1 expounds on the advantages and disadvantages of some metal removal processes.

The DMP set-up consists of three phases, namely, the donor phase, which contains the ion of interest for recovery, the sweep phase, which contains the donating ion to enable the counter transport and, most importantly, the IEM, which controls and allows selective transport of the ions. Cation exchange membranes (CEMs) are used for removing, recovering, separating and concentrating metal ions. Anion exchange membranes are applied during specific treatment of harmful anions such Cl^−^, F^−^, HCO_3_^−^, NO_3_^−^, SO_4_^2−^ and AsO_4_^3−^ [47].

The current work was inspired by the lack of a framework and methodological based analysis that extracts the various DMP phases for the selective recovery and removal of metal ions. In this context, the simplified review looks at the DMP set-up and its main features, DMP application areas, studied factors and research approach in the treatment of metal ions from the wastewater treatment settings. Again, the paper gives a succinct overview of process integration of DMP and other processes for the treatment of metal ions. The components considered in this review process give a perspective to future researchers on the methodological approach to DMP.

## 2. Ion Exchange Membranes (IEM)

**Figure 1:**
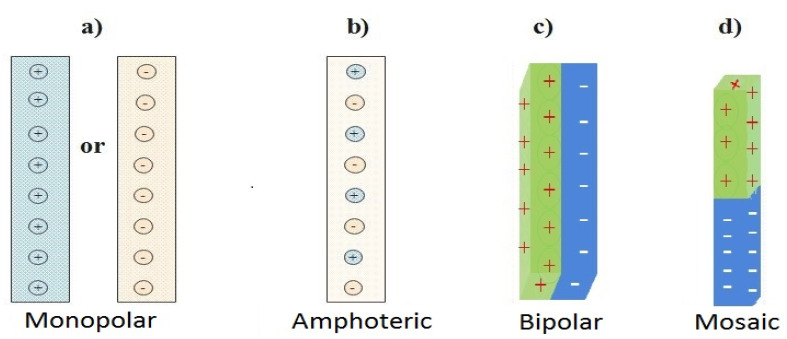
Categorized ion exchange membranes. (**a**) Positive or Negatively charged monopolar IEM, (**b**) Amphoteric IEM, (**c**) Bipolar IEM and (**d**) Mosaic IEM adapted from [49,50].

Monopolar, amphoteric, bipolar and mosaic are the four (4) types of IEMs based on their charge functional groups and fixed ionic group pattern. Most IEMs for commercial applications are identified as monopolar with a single-line pattern [48,49,50]. Figure 1 is a schematic diagram for the classification of IEMs.

Depending on the charge group interconnection on the matrix phase of the membrane structure, IEMs are identified as homogenous and heterogeneous with varying properties and process advantages. In a homogeneous membrane, charged groups are bonded to a polymer backbone, while in a heterogeneous membrane, the ion exchange material is mixed with the polymeric matrix without chemical bonds between them [51,52,53,54].

Homogeneous IEMs have higher conductivity, perm selectivity and a more balanced distribution of functional sites, but they are more costly to produce and have more complex manufacturing phases. Comparatively, heterogeneous IEMs have better chemical stability and mechanical properties over the homogenous ones [55,56]. However, the low electrochemical properties of the heterogeneous IEMs are associated with ionic mobilization pathways, leakage of co-ions in the solution phase and the availability of inert fractions [52].

**Figure 2:**
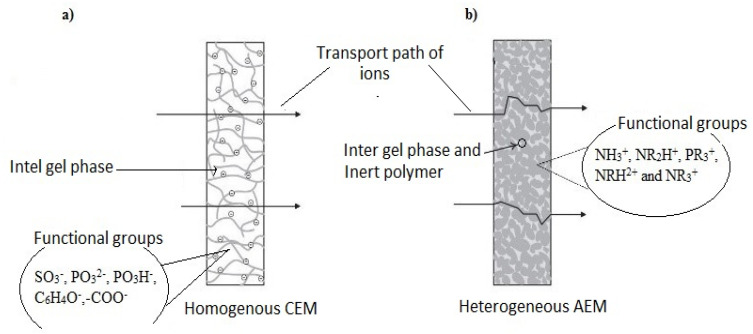
Ions pathway through a homogeneous CEM (**a**) and heterogeneous AEM (**b**).

IEMs are designed and produced to have desirable characteristics such as high permselectivity, high conductivity, good mechanical strength, structural stability and high chemical and thermal stability [57,58,59]. The characteristics are also dependent on factors such as size of the ion exchange resin, resin loading, resin distribution, polymer used, solvent and method. Cation exchange membranes (CEMs) have proven higher stability in strong alkaline solutions than Anion exchange membranes (AEMs). Until recently, most commercially available CEMs and AEMs were homogeneous; Aciplex, Selemion Femion, Nafion, Fumasep, FKS, Ralex and Neosepta are known IEMs [51,60,61]. Figure 2 illustrates a typical transport pathway of ions through a homogenous CEM (Figure 2a) and heterogeneous AEM (Figure 2b).

Non-commercial membranes are often developed for performance evaluation and comparison with commercial membranes. These membranes are either synthesized or result from structural modification of existing membranes. To develop the surface, permselectivity efficiency and ion exchange capacity (IEC) of any membrane, various preparation and modification techniques are applied, which include phase inversion, irradiation and film etching, microfabrication, film stretching, sintering of powders, track-etching, electro-deposition, sol-gel process and coating (dip coating, in situ polymerization, plasma polymerization, interfacial polymerization) [62,63]. However, surface engineering and modification is focused on the use of solvent-free technologies.

In short, IEM characteristics such as ion conductivity, hydrophilicity and hydrophobicity, ionic properties, embedded ion exchange groups, charge density and membrane-ion-affinity are the foundation for application in various ion exchange processes, which includes DMP [50,64,65]. The selectivity transport functionality of the membrane (characterized by morphology and microstructural variation) for target ions in the midst of multivalent ions influences their choice to achieve various DMP separation objectives. For target metal ions, the CEM (Figure 2a) is used.

The activation of CEMs prior to usage in a DMP system is essential to achieve a high membrane hydration. It ensures the setting up of transport pathways for the permeation of ions. Crucial to the conditioning process is the removal of impurities and factory defects from the surface of the membrane. Immersion and conditioning in acid is commonly adopted by researchers [66].

The sequence of conditions commence with immersion in H_2_O_2_, rinsing in distilled water or boiling water and is proceeded with acid conditioning with HCl, H_2_SO_4_ and/or HNO_3_ at an elevated temperature of ≤90 °C [66,67,68]. The treatment chain is then completed by final rinsing in either deionized water at high or normal temperature. However, most treatments do not opt for HNO_3_ conditioning. Further treatment of the CEMs with 1% dilute HCl for 3 hrs enhances ionic transport by increasing the inter-pore hydration of the membrane. Other procedures use NaOH neutralization in between two acid conditioning steps that alternate between HCl and H_2_SO_4_ at different treatment times and temperatures, including room temperature, for the same membrane [69,70].

## 3. Donnan Membrane Cell

Four modules, notably the plate and frame, spiral wound, hollow fiber and the tubular type [71,72,73], are known in the membrane industry. However, two modules are applicable in the DMP system as there is the requirement of separate solutions flowing on either side of the membrane for counter exchange of the ions. These are the plate and frame and the tubular modules. The plate and frame modules are one of the earliest in the membrane industry and consist of a flat sheet membrane and a mesh spacer sandwiched between two blocks and plates. The tubular module consists of smaller tubular compartment housing membranes that are fitted into a larger tube [74]. Flat sheet modules have low performance characteristics, while tubular modules have medium performance characteristics, based on performance parameters such as promoting high cross flow rate, high filtering area to volume packing ratio and a pre-treatment requirement.

**Figure 3:**
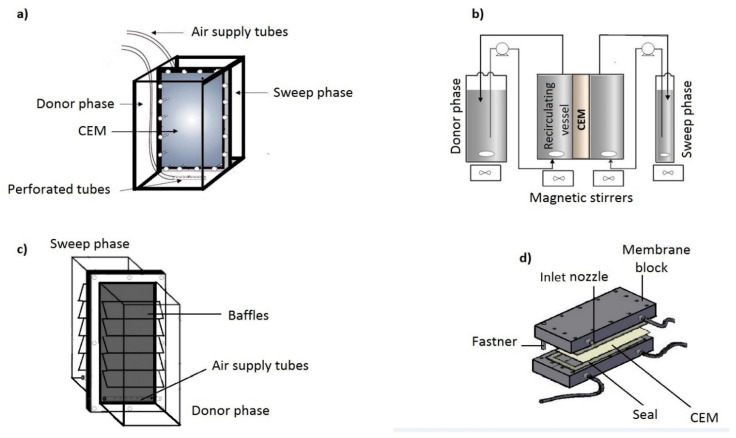
Donnan Membrane Process Cell Designs: (**a**) a simple compartment with compressed air agitation; (**b**) Compartment with external vessels and a mixing unit; (**c**) Point of Use systems; (**d**) Donnan membrane rig.

Various compartments to contain the donor and sweep phase solutions and membrane have been developed over the years. These modules are designed to meet main design criteria cited by [17] for DMP. These compartments are mostly made from materials such as borosilicate glass, Plexiglas (C_5_O_2_H_8_)_n_, PVC (C_2_H_3_Cl)_n_ and Teflon (C_2_F_4_)_n_. A simple two-compartment cell has seen development with the attachment of external donor and sweep side vessels. Flow patterns are set-up with compressed air (Figure 3a), magnetic stirrer (Figure 3b) and shaking blocks or baffles (Figure 3c). In addition, turbulence at the membrane solution surface can be caused by increasing the flow rate of the electrolytic solution for a DMP compartment in Figure 3d. Zhao et al. [75] used a similar set-up as demonstrated in Figure 3c and called it a point of use dialyzer. Additionally, cell arrangements vary and hybrid structures have included a 20 cell pair mounted with CEMs, 11 cells consisting of 5 feed and 6 sweep cells and a 3–4 membrane cell [76,77,78].

### Transport Mechanism

**Figure 4:**
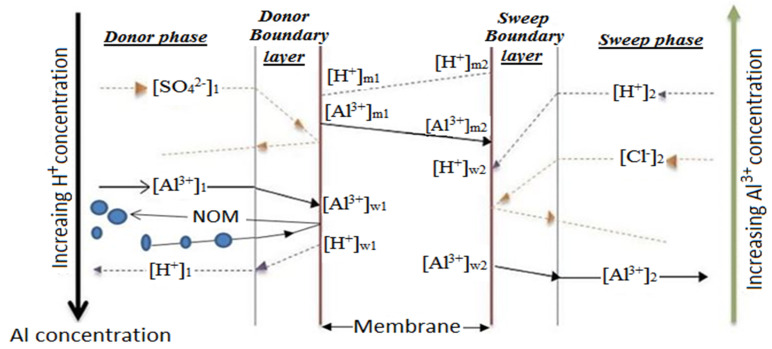
Al^3+^-H^+^ transport through a cation exchange membrane.

In 1924, F.G Donnan discovered the Donnan equilibrium from the electrostatic repulsion of co-ions by the IEM. When using alum, the donor and sweep phases may contain the electrolytic solutions of aluminum sulfate from a potable water treatment residue (PWTR) and hydrochloric acid, respectively. At a level of polarization, dissociation of the acidic salt occurs at the membrane-solution interface and exchange commences. Figure 4 illustrates the ideal exchange mechanism of aluminum and hydrogen ions through a CEM. As the counter transport occurs, a concurrent electrostatic exclusion of sulfate and chloride co-ions by the Coulomb forces of the fixed ions in the membrane matrix also takes place [79]. From Figure 4, the counter transport of Al^3+^-H^+^ is a three-stage process that involves the convective mass transfer of Al^3+^ from the donor solution (1) to the solution–membrane interface (w1) and the diffusion of the metal ion through the ion exchange membrane donor side to the membrane sweep side (m1 to m2). This is followed by convective mass transfer of the ions from the membrane solution interface (w2) to the sweep solution (2).

Since the ion transport is time dependent, the exchange continues until chemical potential gradient equilibrium and counter ion transport equilibrium is reached when electroneutrality is established between the electrolytic solutions. Consequently, the aluminum in the donor solution decreases. The ion of interest is now recovered in a concentrated form in the sweep solution. The PWTR solution, which contains aluminum sulphate, would then be recovered as aluminum chloride. The aluminum chloride can be used as a coagulant. The significance of aluminum chloride recovery eventually leads to recycling, re-use and purification.

The entropic gain by the exchanging of the monovalent metal ion and the trivalent metal ion can be expressed by the Donnan potentials of each metal specie. Generally, the Donnan potential for each metal specie (*i*) by their concentrations in each phase of the membrane [80,81] is expressed as

(1) EDon=RTFIn[ai,dai,s]1z

where *E_Don_* is the Donnan potential, *R* is the gas constant (8.314 J/kmol), *F* is the Faraday constant (96485 C/mol), *T* is the temperature (K), *z* is the valence of the metal ion and *a_i,d_* and *a_i,s_* are the activity of the metal ions in their respective phases.

When the counter exchange of the metal strives towards equilibrium, concentration of the metal ions is not equal; rather, the ratio of the valance of the metal ions to the power one equilibrates [82,83]. The equilibration theory of the metal species in both phases, in a typical case of the transport between trivalent aluminum and monovalent hydrogen, will be:

(2) [aAl3+,daAl3+,s]13=[aH+,daH+,d]1

## 4. Trends for Target Metal Ion

The Donnan membrane process applications cover various industries spanning from the mineral process to the water and wastewater treatment industry. Depending on the DMP configuration, operating variables that affect recovery, separation and concentration of target metal ions are the concentration and flowrates of donor and sweep phases, electrolytic sweep solution, valence of counter ion, pH, experimental duration, membrane type and morphology [84].

Most researchers use the one-factor at a time (OFAT) approach to evaluate the transport of metal ions. In OFAT, one factor is varied while the other variables are kept constant. Using OFAT, multiple experiments cannot be run, while a high number of experiments makes it cost intensive and time and resource consuming, with the inability to assess the interactive effect of variable optimal settings [85,86,87].

The statistical approach, also known as design of experiment (DOE), allows researchers to evaluate the independent and interacting effect of various process variables under consideration. Therefore, statistical models were developed that aid in process optimization [88,89,90].

Two relevant polynomial models are often involved. The first model, as seen in Equation (3), is for special cases, and this includes first-degree models (d = 1). The second degree model (d = 2) is also expressed in Equation (4) [91] as:

(3) $$Y=\beta_{o}+\overset{k}{\underset{i=1}{\sum }}\beta_{i}x_{i}+\epsilon$$

(4) $$Y=\beta_{0}+\overset{k}{\underset{i=1}{\sum }}\beta_{i}x_{i}+\sum_{i<j}\sum \beta_{ij}x_{i}x_{j}+\overset{k}{\underset{i=1}{\sum }}\beta_{ii}x_{i}^{2}+\epsilon$$

where *Y*, $\beta_{0}$, $\beta_{i}$, $x_{i}$ and ϵ are the characteristic response, constant term, coefficient, independent process variable and random experimental error at a zero mean, respectively.

A statistical approach has been used in only a few DMP studies involving target metal ions to assess the impact of process variables on recovery. A face centered central composite model developed for Al^3+^ considered the donor phase concentration, donor phase flowrate, sweep concentration and sweep flowrate [92]. Furthermore, screening studies for the four factors indicated that the sweep concentration had an insignificant effect on aluminum recovery [93]. As such, a Box–Behnkein model was developed using the donor concentration, donor flowrate and sweep concentration as factors for the design matrix.

Kinetic models have been developed for monovalent (Na^+^, K^+^, Cs^+^ and Ag^+^), divalent (Ca^2+^, Ba^2+^, Mg^2+^ and Sr^2+^) and trivalent (Al^3+^) metal ions based on Fick’s and Nernst–Planck’s equations for ion fluxes [94,95,96,97,98,99]. Interestingly, all kinetic models for the mass transfer of the metal ions through the membrane have been conducted using the Nafion 117 membrane. Different commercially available Nafion membranes for possible DMP studies and their respective properties are presented in Table 2 [100,101,102,103,104,105,106,107].

### 4.1. Single Stage

Laboratory scale experiments for the recovery and removal of metal ions are reviewed in a single stage DMP process as shown in Table 3. Most of the donor phase solutions from industrial streams required pre-treatment such as acidification and filtration prior to DMP.

### 4.2. Multi-Treatment Technologies

To achieve synergic advantage in target metal ion separation, recovery and concentration, individual process limitations must be resolved.

The DMP process has been used as a possible pre-treatment for the removal of ion inhibitors, fouling and scaling sediments and as a post-treatment to further remove target ions. Table 4 consists of two stage combinative processes for the recovery. The Donnan membrane process has been integrated in three or more multi-stage processes such as the recycling of the lithium ion battery [115] and recovery of Fe from Fe-PWTR by integrating DMP with recovery and purification technologies such as acid leaching, adsorption with activated carbon, ultrafiltration and caustic treatment [20].

DMP in reverse osmosis (RO) and ion exchange (IEX) application studies looked into regeneration of resins using RO brines as the sweep phase with DMP as the regeneration step [78,116]. In using DMP as a pretreatment to RO, the performance is affected by monovalent to multivalent ion ratio in the feed stream [78].

Additionally, DMP as a pretreatment in ED and reverse ED would change the ionic composition of the feed solution, thereby increasing the limiting current density of the solution before desalination takes place, and also reducing scaling [76,83]. While a CEM or AEM would be considered for the DMP in a DMP-RO system, Rózańska and Wiśniewski [117] used an integrated system made up of Selemion-CMV and Neosepta AFN, which are CEM’s and AEM’s, respectively.

Furthermore, the introduction of DMP in coagulation established that coagulant application at the sweep side provided extra driving ions for counter transport of target ions [118]. Table 4 shows the application of DMP for the treatment of target metals.

### 4.3. Future Prospects

The Donnan membrane process, unlike the pressure-driven membrane process, is a potential gradient process that is caused by concentration and the driving force. However, over 95 years after the inception of DMP, the technology is yet to be fully researched and have its full potential understood. As an extremely slow kinetic process for ion transport that takes a longer period to achieve equilibrium, and concentration and separation of metal species, many approaches have not geared towards industrial applications [122,123]. Therefore, there is the proposed need for the integration of DMP to other separation processes rather than have a single DMP system, as cited in Table 2, where DMP is incorporated with other processes.

Another possible deterrent to DMP use is the membrane’s higher purchase price and variable selectivity [124]. However, this is the situation for every emerging technology as initial purchase affects the total expenditure cost. Research and development are, therefore, geared towards addressing such problems. The cost will decline when global demand soars with progress in research and development towards cheaper membrane production costs.

There is little knowledge in the literature about factors like donor flowrate, electrolytic sweep solution flowrate, pH and experimental length, as well as their synergistic effect on separation, recovery and concentration of target metal ions. As a consequence, future research is needed. The various documented research studies do not make wide and industrial realistic variations to these process conditions. Additionally, understanding of the selectivity of IEM with multi-ion solutions should be given attention, as CEMs might be known to permeate target ion;, however, the transport of non-targeted ions would occur in comparatively low to high concentrations.

## 5. Conclusions

The deployment of the Donnan membrane process for separation, recovery and concentration of metal ions is feasible with recommendable performance. This work attempted to give a succinct account of the DMP on target metal ions, which includes the cells, IEM, applications and treatment outcomes. This approach provides a quick referencing opportunity for expanding the prospects of DMP on target metal ions. For instance, the propensity of DMP to selectively recover aluminum whilst rejecting organics places it a step ahead over other techniques for recovering metals, specifically heavy metals. Consequently, stakeholders investing in Donnan membrane technology with real-time monitoring in metal ion treatment are poised to provide significant opportunities for socio-economic growth and development.

## Figures and Tables

**Table 1:** Advantages and disadvantages of selected metal removal technologies.

Process	Advantages	Disadvantage	References
Conventional Ion exchange	Low cost, high selectivity, little or no use of organic solvents, regeneration capability	Resin regeneration requires chemical addition, poor quality products, long production cycle, finding suitable resin is a challenge, process is highly pH sensitive.	[9,22]
Pressure driven membranes	Wide range application, simple configuration, high removal and rejection.	Susceptible to fouling, complex reverse cleaning process, additional pretreatment process is costly, internal and external concentration polarization depending on membrane process, expensive and non-recyclable drawing solutions for forward osmosis process, enrichment of contaminant in retentates causing secondary pollution, non-rejection of monovalent ions for nanofiltration, high energy demand for pressure pumps used.	[23,24,25,26,27,28]
Adsorption	Simple technology, wide range of metals selectivity, low cost local, materials readily available as natural absorbents,	High cost of absorbent, residue generation and disposal challenges, adsorbent regeneration complex and expensive, pH of solution affects sorption to binding sites, removal efficiency depends on type of sorbent, synthetic absorbent expensive to produce.	[29,30,31,32]
Chemical precipitation	Simple, low cost of precipitant, non-selective, shorter removal time.	pH adjustment is critical as precipitates can resolubilize, high residue generation and disposal, high chemical demand, large tanks at high installation costs, energy inputs required, generation of H_2_S for sulfide reagent, CO_2_ for carbonate reagent.	[33,34,35,36]
Bioremediation	Moderate cost, no waste generation, minimum or no disturbance to the soil, no ecosystem disruption, minimal energy requirement, large contaminants handled at a time.	Not recommended for non-biodegradable compounds, products after biodegradation can be more toxic, problematic upgrading from laboratory scale, contaminant migration through environmental resources, time consuming process, remobilization of stabilized contaminants due to changes in hydrological and geochemical conditions, inadequate benchmark values for field application, requires deep understanding of microbial process.	[37,38,39,40,41]
ED/reverse ED	Ion transport is rapid, effective in wide pH ranges, no phase change, not affected by osmotic pressure.	Stack clogging and membrane fouling, high energy consumption, skilled labor, compatibility of membrane and stacks materials to feed stream solution is highly required, current density limit, requires post treatment and pretreatment.	[42,43,44,45,46]

**Table 2:** Commercially available Nafion membranes with their respective properties.

Nafion	Formation	Equivalent Weight (g eq^−1^)	Nominal Thickness (µm)	Basic Weight (g m^−2^)
N 115	Extrusion	1100	127	250
N 117		1100	178–183	360
N 1035		1000	89	175
NR 212	Solution casted	1100	50–51	100
NR 211		1100	25.4	50
XL	Reinforced	1100	27.5	55
HP		-	20	43.5
424		1100	180	540
1110	Extrusion	1100	254	500

**Table 3:** Target metal ion recovery using DMP only.

Metal	Stream	Phase Conditions			IEM	Highlights	Reference
		Volume (Donor:Sweep) Ratio	Donor pH	Sweep Condition			
Al^3+^	PWTR	4:1	3–3.5	1–2 M H_2_SO_4_	Nafion 117 (HM) Ionac 3470 (HT)	%R (Al^3+^) Ionac was 55% < %R (Al^3+^) Nafion 117. Trace permeation of Fe^3+^, Zn^2+^, Cu^2+^ and As^3+^.	[13,81]
Ti^4+^, Fe^3+^, Al^3+^, Na^+^	Bauxite waste	-	0.7–0.1	0.05–1 M HCl	Neosepta CMB (HM) Neosepta CMX (HM) ICE 450-SA_3_T (HT) ICE 450-SA_3_S (HT)	Fluxes for all membranes follow the order Fe^3+^ > Al^3+^ > Na^+^ > Ti^4+^. Recovery in all membranes mostly follow the order of Na^+^ > Fe^3+^ > Al^3+^ > Ti^4+^. Chelating agents either increases or decreases transport of metal ion.	[70,108]
Au^+^	Circuit board scrap	1:1	0.84	0.1–4 M NaCl	Micro-pore grafted CEM	%R (Au) = 89% Au with trace transport of Cu and Ni despite being in high mass ratio in the donor phase after 4 cycles of treatment.	[109]
Fe^3+^	PWTR	2:1 4:1	3–3.5	1 M H_2_SO_4_	Nafion 117 (HM) Nafion 115 (HM)	%R (Fe) = 82% at 2:1 against %R (Fe) = 76% at 4:1.	[13,20]
Ca^2+^ and Mg^2+^	PWTR	1:1	-	0.02 M HCl	Nafion 117 (HM)	%R (Ca^2+^) = 20% and %R (Mg^2+^) = 50%.	[110]
Ca^2+^ and Mg^2+^	Tap water	-	6.8–7	0.1 M HCl	Four Modified PVDF membrane	%R (Mg^2+^) = 80% and %R (Ca^2+^) = 70–72%. Modification improved membrane properties, hence better performance than synthesized and unmodified PVDF membrane.	[111]
Cu^2+^ and Ag^3+^	SS	2–10:1	-	1–3 M HNO_3_	Selemion CMV (HT)	Fluxes for Cu^2+^ > Ag^3+^. Selectivity of both cations improve with the insertion of cation exchange textile between the CMV membranes. Cu^2+^ enrichment in sweep solution is 1.5–3.9 while Ag^3+^ was 1.2–7.9.	[112]
Cu^2+^, Co^2+^ and Ni^2+^	SS	2:1	-	0.01 M H_2_SO_4_ (pH 1–4)	ICE 450-SA_3_T (HT) ICE 450-SA_3_S (HM) Spectrapor Dialysis membrane	Flux of metal ions decreases with increasing pH of sweep solution for ICE membranes and vice versa for spectrapor. Recovery of metal ions by membrane is SA_3_S > SA_3_T > Spectrapor	[113]
Cr^3+^ and Cu^2+^	SS	-	3	0.1 M HCl	Four different PVDF/P2FAn composite membrane synthesized with dopants	Flux and recovery of Cu is higher than Cr due to smaller hydration volume. Dopant effect on Cr recovery was similar for NSA and PTS. Dopant effect on Cu recovery was SDS > ABS > PTS > NSA.	[114]

Toluenesulfonate, NSA—1,3 (6 or 7)-naphthalene trisulfonic acid; ABS—o-aminobenzen sulfonic acid; SDS—sodium dodecyl sulfate; P2Fan—poly2-fluoroaniline; SS—synthetic solution; PWTR—Potable water treatment residue.

**Table 4:** Donnan membrane process and other treatment technologies.

Combined Process	Target Metal/Ion	Feed Phase for DMP	Comments	Reference
Electrodialysis	Ca^2+^ and Mg^2+^	Brackish solution	Desalination increased by 21% with observed reduction in energy consumption after 79–89% Ca^2+^ and 75–90% Mg^2+^.	[76,117,119,120]
Reverse electrodialysis	Ca^2+^ and Mg^2+^	River and sea water	Gross and net power density improved by 1.4–9% and 6.3%, respectively.	[83]
Reverse Osmosis	Ca^2+^ and Mg^2+^	Potato Processing waste water and Tap water	DMP increases RO treatment by 16% and 47% more for wastewater and tap water.	[78]
Struvite	Zn^2+^, K^+^, Na^+^, Mg^2+^ and Fe^3+^	Hydrolyzed sludge liquid	Struvite composition met regulatory requirement as DMP recovery of metal composition was high.	[121]

## Data Availability

Data sharing not applicable.
